# 3D Printing in Wound Healing: Innovations, Applications, and Future Directions

**DOI:** 10.7759/cureus.75331

**Published:** 2024-12-08

**Authors:** Rahul A Sachdeo, Chitra Khanwelkar, Amol Shete

**Affiliations:** 1 Department of Pharmacology, Krishna Institute of Medical Sciences, Krishna Vishwa Vidyapeeth (Deemed to Be University), Karad, IND; 2 Department of Pharmaceutics, Krishna Institute of Pharmacy, Krishna Vishwa Vidyapeeth (Deemed to Be University), Karad, IND

**Keywords:** ‎3d printing, bioprinting, personalized medicine, skin substitutes, tissue engineering

## Abstract

The field of wound healing faces significant challenges, particularly in the treatment of chronic wounds, which often result in prolonged healing times and complications. Recent advancements in 3D printing technology have provided innovative solutions to these challenges, offering tailored and precise approaches to wound care. This review highlights the role of 3D printing in enhancing wound healing, focusing on its application in creating biocompatible scaffolds, custom wound dressings, and drug delivery systems. By mimicking the extracellular matrix (ECM) and facilitating cell proliferation, 3D-printed biomaterials have the potential to significantly accelerate the healing process. In addition, 3D bioprinting enables the production of functional skin substitutes that can be customized for individual patients. Despite the promise of these technologies, several challenges remain, including the need for improved vascularization, cost concerns, and regulatory hurdles. The future of wound healing lies in the continued integration of 3D printing with emerging technologies such as 4D printing and bioelectronics, providing opportunities for personalized and on-demand therapies. This review explores the current state of 3D printing in wound care, its challenges, and the future potential of these innovative technologies.

## Introduction and background

Wound healing is a complex biological process involving a series of orchestrated events, including hemostasis, inflammation, proliferation, and tissue remodeling [1]. While acute wounds generally progress through these stages successfully, chronic wounds such as diabetic ulcers, venous leg ulcers, and pressure sores present significant challenges due to delayed or impaired healing [2]. Chronic wounds are increasingly prevalent and often lead to severe complications such as infections, amputations, and diminished quality of life [3]. Conventional wound care methods, including dressings, skin grafts, and topical treatments, frequently fall short of addressing the complexities of these wounds, highlighting the need for innovative solutions to enhance tissue regeneration and promote effective healing [4,5].

In recent years, 3D printing has emerged as a transformative technology in the biomedical field, offering unique advantages in designing and fabricating complex, patient-specific medical devices and tissues [6]. Initially developed for industrial applications, 3D printing has been adapted for tissue engineering and regenerative medicine, holding great promise for improving wound healing [7]. This technology facilitates the creation of custom, biocompatible scaffolds, wound dressings, and skin substitutes tailored to individual patient needs. Additionally, 3D bioprinting allows for the precise deposition of cells and biomaterials, closely mimicking the ECM to provide structural support and promote cell proliferation [8].

The potential of 3D printing to overcome the limitations of traditional wound care is substantial. Applications include personalized skin grafts for burn victims and drug-loaded scaffolds for controlled drug release [9]. This review aims to explore the current state of 3D printing technologies in wound care, highlight recent advances in 3D-printed scaffolds and skin substitutes, and discuss the future potential of this revolutionary technology. By examining the integration of 3D printing in wound healing, this paper seeks to provide insights into how personalized medicine and regenerative therapies can reshape wound care practices [10,11].

Current challenges in wound healing include delayed healing in chronic wounds, infection management, and the inability to fully regenerate complex tissue structures. Additionally, factors such as aging, comorbidities like diabetes, and the rise of antibiotic-resistant infections further complicate effective wound management. Wound healing is a complex biological process comprising several stages: hemostasis, inflammation, proliferation, and remodeling [12]. Each stage plays a critical role in restoring tissue integrity and function. 

The initial hemostatic response involves the formation of a clot to prevent excessive blood loss and the release of growth factors that initiate tissue repair. This is followed by the inflammatory phase, where immune cells clear debris and pathogens, setting the stage for tissue regeneration. The proliferation phase is characterized by the formation of new tissue through processes such as angiogenesis, fibroplasia, and re-epithelialization. Finally, the remodeling phase involves the maturation and reorganization of the ECM, which strengthens the newly formed tissue and restores normal tissue function [13].

Despite these well-orchestrated phases, chronic wounds such as diabetic ulcers, pressure sores, and venous leg ulcers present significant challenges [14]. These types of wounds often fail to progress through the normal healing stages, resulting in prolonged inflammation, impaired tissue regeneration, and persistent pain. Chronic wounds are frequently complicated by factors such as poor blood circulation, infection, and underlying health conditions like diabetes, which further impede the healing process [15]. For instance, diabetic ulcers are characterized by impaired wound healing due to factors like neuropathy, poor glycemic control, and reduced immune response [16]. Pressure sores, also known as pressure ulcers, result from prolonged pressure on specific areas of the body, leading to tissue ischemia and necrosis [17].

Traditional wound care techniques, including standard dressings, topical treatments, and skin grafts, often fall short of addressing the complexities of chronic wounds. Conventional dressings may not adequately manage exudate, provide an optimal healing environment, or support tissue regeneration [18]. Skin grafts and other advanced wound care products, while effective in some cases, may not be suitable for all patients due to factors such as graft rejection, limited availability, and high costs [19]. Additionally, these methods often lack the ability to address the underlying causes of chronic wounds, such as poor circulation or chronic inflammation [20]. As shown in Table 1, a comparison of the limitations of conventional wound care versus the benefits of 3D printing technologies highlights the significant advantages offered by 3D printing in terms of customization, efficiency, and enhanced healing outcomes.

**Table 1:** Limitations of Conventional Wound Care Versus the Benefits of 3D Printing Technologies

Aspect	Conventional wound care: limitations	3D printing technologies: benefits
Customization	Standardized products; limited ability to tailor to individual needs	Highly customizable wound dressings and scaffolds
Healing efficiency	May not fully support complex wound structures or chronic wounds	Enables precise anatomical and functional healing
Material utilization	May use materials that are non-biodegradable or poorly biocompatible	Uses biocompatible and biodegradable materials
Cost-effectiveness	Long-term costs may be high due to repeated use of standard products	Potential for cost-effective solutions tailored to needs
Infection control	Limited ability to incorporate antimicrobial or bioactive agents	Facilitates incorporation of antimicrobial/bioactive agents
Innovation and versatility	Restricted by manufacturing constraints	Enables novel designs like smart dressings and bioinks
Production speed	Mass production but lacks flexibility for rapid prototyping	On-demand production and rapid prototyping capabilities
Integration with technology	Minimal use of advanced monitoring or diagnostics	Can integrate sensors and smart systems for real-time data

There is a growing need for innovative solutions that can enhance wound healing by addressing these limitations. Advanced wound care technologies, including 3D-printed scaffolds, bioengineered skin substitutes, and controlled drug delivery systems, offer promising alternatives to traditional methods [21]. These innovations aim to provide customized, patient-specific treatments that can better manage wound exudate, deliver therapeutic agents directly to the wound site, and promote more effective tissue regeneration [22]. By overcoming the limitations of conventional approaches, these advanced solutions hold the potential to significantly improve outcomes for patients with chronic wounds.

This review aims to provide a comprehensive overview of advancements in 3D printing technologies and their applications in wound healing, emphasizing innovations, current applications, and future directions. To ensure a thorough and balanced discussion, a structured search strategy was employed, utilizing recent databases available. This approach highlights the scope of the review and its contribution to advancing the understanding of 3D printing in regenerative medicine.

## Review

**Figure 1:**
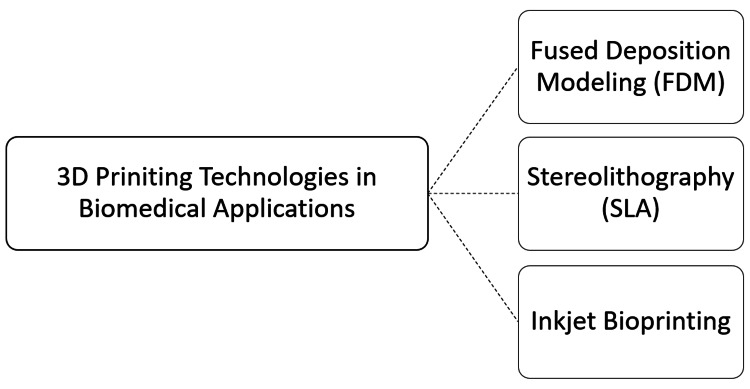
Overview of 3D Printing Technologies in Biomedical Applications The image was illustrated by the author Rahul Sachdeo.

3D printing, also known as additive manufacturing, has rapidly advanced in recent years, offering transformative possibilities in biomedical applications. The technology encompasses several methods, each with distinct advantages and applications in creating complex structures for wound healing. The most commonly used 3D printing technologies include fused deposition modeling (FDM), stereolithography (SLA), and inkjet bioprinting. Figure 1 illustrates 3D printing techniques. These technologies enable the fabrication of complex structures for personalized medicine, tissue engineering, drug delivery systems, and regenerative medicine, providing innovative solutions to create biocompatible scaffolds, implants, and prosthetics tailored for specific medical needs (Figure 1).

FDM is a widely used 3D printing technique that involves the extrusion of thermoplastic polymers through a heated nozzle. The material is deposited layer by layer to build the desired structure. FDM is valued for its simplicity, cost-effectiveness, and versatility, making it suitable for fabricating scaffolds and other structures in biomedical applications [23]. However, FDM's resolution and material choices are limited compared to other techniques.

SLA uses a laser to polymerize a photosensitive resin, solidifying it layer by layer to create precise and high-resolution structures. SLA is known for its high accuracy and surface finish, making it ideal for producing detailed models and complex geometries required in tissue engineering [24]. The main limitations of SLA include the requirement for post-processing and the relatively higher cost of the materials used.

Inkjet bioprinting is an advanced technique that involves the deposition of biological materials, such as cells and bioinks, onto a substrate to create complex tissue structures. This method allows for high precision and the incorporation of living cells into the printed constructs, which is crucial for creating functional tissue models and promoting cell viability and tissue integration [25]. The primary challenges of inkjet bioprinting include ensuring cell survival during the printing process and achieving consistent biocompatibility with various bioinks. As illustrated in Table 2, various 3D printing technologies offer distinct advantages in wound healing applications, with differences in materials, key features, and specific therapeutic uses, showcasing their potential to revolutionize wound care.

**Table 2:** Comparison of 3D Printing Technologies for Wound Healing: Materials, Features, and Applications

3D Printing Technology	Materials Used	Key Features	Applications in Wound Healing	Advantages	Limitations
Fused deposition modeling (FDM)	Thermoplastic polymers (e.g., PLA, PCL, TPU)	Layer-by-layer deposition, simple, and cost-effective	3D-printed wound dressings, scaffolds for tissue regeneration	Cost-effective, rapid prototyping, customizable shape and size	Limited material options, less resolution for fine details
Stereolithography (SLA)	Photopolymers, hydrogels, biocompatible resins	High-resolution, UV light curing of resin-based materials	High-precision 3D-printed scaffolds, wound dressings with complex structures	High precision, smooth surface finish, suitable for creating fine details	Expensive, limited material types, slower print speeds
Inkjet printing	Bioinks (cell-based, growth factors, hydrogels)	Deposition of liquid droplets onto substrates, can print cells and biomaterials	Bioprinted skin substitutes, drug delivery systems for wound treatment, smart wound dressings	Precise control over droplet placement, capable of printing cells and bioactive materials	Requires bioinks, complex post-processing, limited scalability for larger structures

**Figure 2:**
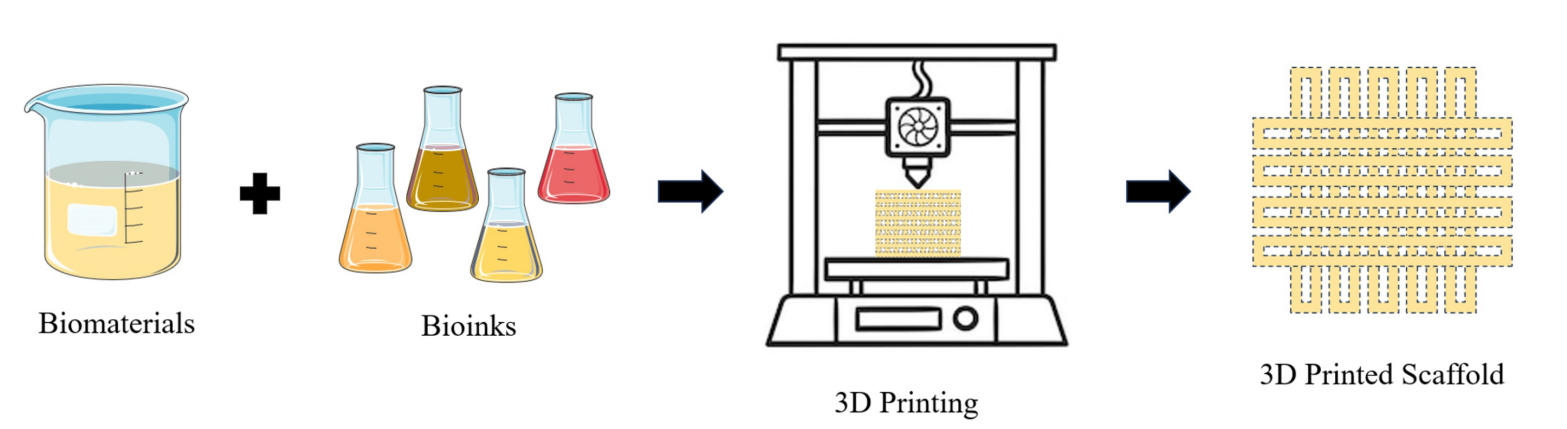
Workflow of 3D Bioprinting The image was illustrated by the author Rahul Sachdeo

The materials used in 3D printing for biomedical applications are diverse, encompassing biodegradable polymers, hydrogels, and bioinks. Biodegradable polymers such as polylactic acid (PLA) and polycaprolactone (PCL) are commonly used due to their ability to degrade safely within the body and their suitability for various scaffold designs [26]. Hydrogels are water-swollen networks of polymer chains that offer a moist environment conducive to cell growth and tissue regeneration. They are particularly useful for creating tissue-like structures and delivering therapeutic agents directly to the wound site [27]. Bioinks, which consist of living cells suspended in a gel matrix, are crucial for fabricating functional tissue constructs and ensuring that the printed structures can integrate with host tissues [28]. The workflow of 3D bioprinting is depicted in Figure 2.

The key advantages of 3D printing in wound healing include its ability to create highly customized and patient-specific structures. This technology allows for the precise fabrication of scaffolds and skin substitutes that match the unique dimensions and needs of individual wounds. Furthermore, 3D printing enables the integration of multiple materials and functionalities within a single construct, such as incorporating growth factors or antimicrobial agents directly into the scaffold. By providing tailored solutions and supporting complex tissue architectures, 3D printing represents a significant advancement in wound care and regenerative medicine [29].

3D-printed scaffolds for wound healing

3D-printed scaffolds have emerged as a promising solution for enhancing wound healing by providing a supportive framework that mimics the natural ECM. The design of these scaffolds is crucial in promoting effective tissue regeneration and facilitating the repair of damaged tissues.

Design of Biocompatible Scaffolds

The primary goal in designing 3D-printed scaffolds is to create structures that closely replicate the ECM, which is essential for cellular attachment, proliferation, and differentiation. The ECM provides not only mechanical support but also biochemical signals necessary for tissue regeneration. Advanced 3D printing technologies allow for the precise control of scaffold architecture, including pore size, interconnectivity, and mechanical properties, to match the characteristics of the native ECM [30]. By achieving such fidelity, these scaffolds can better support cell growth and tissue integration, leading to improved healing outcomes.

Biomaterials for Scaffold Creation

Various biomaterials are employed in the creation of 3D-printed scaffolds, each offering unique advantages. Collagen, a key structural protein in the ECM, is widely used due to its biocompatibility and ability to support cellular attachment and proliferation [31]. Gelatin, a derivative of collagen, is also utilized for its similar properties and its ability to be easily modified to suit different scaffold designs [32]. Chitosan, a biopolymer derived from chitin, is favored for its biodegradability, antibacterial properties, and ability to support cell growth and tissue regeneration [33]. The choice of biomaterial depends on the specific requirements of the wound site and the desired properties of the scaffold.

Functionalization of Scaffolds

To further enhance the effectiveness of 3D-printed scaffolds, they can be functionalized with growth factors and bioactive molecules. Growth factors, such as epidermal growth factor (EGF) and vascular endothelial growth factor (VEGF), are critical for stimulating cellular activities such as proliferation, migration, and angiogenesis. Incorporating these factors into scaffolds can accelerate wound healing and improve tissue repair. Additionally, bioactive molecules, including antimicrobial agents and anti-inflammatory compounds, can be integrated to address specific issues such as infection and chronic inflammation [34]. This functionalization not only improves the scaffold's performance but also provides a more comprehensive approach to managing complex wound healing challenges.

The combination of advanced scaffold design, choice of biomaterials, and functionalization strategies represents a significant advancement in wound care. By leveraging these approaches, 3D-printed scaffolds offer a tailored solution that enhances the natural healing process and supports the regeneration of damaged tissues [35].

3D bioprinting of skin substitutes

3D bioprinting has revolutionized the development of skin substitutes, offering significant advancements in creating functional and personalized skin models. This technology allows for the precise deposition of living cells and biomaterials to produce skin substitutes that can effectively address various types of wounds.

Advances in Bioprinting Full-Thickness Skin Models

The development of full-thickness skin models using 3D bioprinting involves the integration of various skin cells, including fibroblasts, keratinocytes, and other specialized cell types. Fibroblasts are essential for producing the dermal matrix, which provides structural support and aids in collagen synthesis [36]. Keratinocytes, the predominant cells in the epidermis, are crucial for forming the outer protective layer of the skin and ensuring barrier function [37]. Recent advances in bioprinting techniques have enabled the precise layering of these cells, along with the incorporation of additional elements such as endothelial cells and melanocytes, to create more complex and functional skin substitutes [38]. These advancements enhance the ability to model the skin’s natural architecture and improve the integration of the substitutes with the host tissue.

Role of 3D-Printed Skin Substitutes in Chronic and Burn Wound Treatment

3D-printed skin substitutes play a crucial role in the treatment of chronic wounds, such as diabetic ulcers and pressure sores, as well as burn injuries. Chronic wounds often exhibit delayed healing and require specialized treatments to address underlying issues such as impaired circulation and persistent inflammation [39]. 3D-printed skin substitutes offer a customized solution by providing engineered tissue that can mimic the properties of healthy skin and support the regeneration of damaged tissues. In burn wound treatment, these substitutes can be used to cover extensive areas of injury, providing a protective barrier and promoting the healing of both superficial and deep burns [40]. The ability to customize the design and composition of the skin substitutes ensures that they meet the specific needs of each patient, improving overall treatment outcomes.

Personalized Skin Grafts

One of the most significant advantages of 3D bioprinting is the ability to create personalized skin grafts tailored to individual patients. This customization involves designing skin substitutes that match the patient’s unique wound characteristics, such as size, depth, and tissue type. By using patient-specific cells and materials, personalized skin grafts can improve graft integration, reduce the risk of rejection, and enhance the overall effectiveness of the treatment [41]. Additionally, the incorporation of patient-specific data into the bioprinting process allows for the development of skin substitutes that address specific medical conditions, such as genetic skin disorders or chronic wounds. This tailored approach represents a significant advancement in personalized medicine and offers a more precise and effective solution for wound healing.

The integration of 3D bioprinting technology into skin substitute development has the potential to transform wound care by providing highly functional, personalized, and effective treatment options. As technology continues to advance, it is expected to play an increasingly important role in addressing the challenges associated with chronic and burn wounds [42].

Applications of 3D printing in chronic wound care

3D printing technology has significantly advanced the field of chronic wound care by providing innovative solutions tailored to the specific needs of patients with difficult-to-treat wounds. This technology allows for the creation of customized dressings, patches, and regenerative constructs that address the complexities of chronic wounds.

Innovations in Wound Dressing Design

The design of 3D-printed wound dressings has evolved to include advanced features such as smart dressings with sensors and controlled-release drug systems. Smart dressings incorporate embedded sensors that monitor various parameters, such as temperature, pH, and moisture levels, providing real-time data on the wound environment [43]. This information can be used to adjust treatment protocols and ensure optimal wound management. Additionally, controlled-release drug systems integrated into 3D-printed dressings allow for the localized delivery of therapeutic agents, such as antibiotics or growth factors, directly to the wound site. This approach can reduce the need for systemic medications and target specific issues, such as infection or inflammation, more effectively [44].

Regenerative Therapies Involving 3D-Printed Constructs

In regenerative medicine, 3D-printed constructs have shown great promise in treating non-healing wounds. These constructs include scaffolds and tissue-engineered grafts that support tissue regeneration and repair. Scaffolds are designed to provide a temporary matrix that promotes cell growth and tissue formation while gradually degrading as new tissue is generated. For instance, 3D-printed scaffolds made from biodegradable polymers have been used to support the regeneration of skin and subcutaneous tissues in chronic wound cases. Tissue-engineered grafts can be tailored to match the patient’s tissue characteristics, enhancing graft integration and reducing the risk of rejection. One notable application involved the use of a 3D-printed skin substitute for a patient with a non-healing venous ulcer, which significantly improved wound healing and quality of life.

The integration of 3D printing into chronic wound care represents a significant advancement, offering customized solutions that address the unique needs of patients with complex wounds. By combining innovative dressing designs, smart technologies, and regenerative therapies, 3D printing enhances the effectiveness of wound management and supports better clinical outcomes.

Quantitative data on the success rates and healing time reduction associated with 3D-printed wound care products are still limited but promising. Preliminary studies have shown that 3D-printed wound dressings and scaffolds can significantly reduce healing times, with improvements in tissue regeneration and reduced infection rates compared to traditional treatments. Further clinical trials and data collection are needed to establish robust statistical evidence on the effectiveness and consistency of these advancements.

3D-printed drug delivery systems

3D printing has introduced innovative methods for creating drug delivery systems that provide controlled and sustained release of therapeutic agents at wound sites. This technology enables the precise fabrication of scaffolds and delivery vehicles that enhance the effectiveness of treatments by targeting drug release to specific areas and timeframes.

Development of Drug-Loaded 3D Scaffolds for Controlled and Sustained Release

The development of 3D-printed drug-loaded scaffolds represents a significant advancement in wound care. These scaffolds are designed to deliver drugs in a controlled manner over an extended period, which is crucial for maintaining therapeutic levels at the wound site and promoting effective healing. The incorporation of therapeutic agents, such as antibiotics, anti-inflammatory drugs, and growth factors, into the scaffold matrix ensures that these agents are released gradually, reducing the frequency of administration and minimizing systemic side effects. For example, 3D-printed scaffolds loaded with antibiotics have been shown to effectively combat infections in chronic wounds, while scaffolds with growth factors support tissue regeneration and repair [45]. The ability to tailor the release profiles of these drugs through scaffold design and material selection enhances treatment outcomes and patient compliance.

Use of 3D-Printed Drug Delivery Systems for Localized Delivery

The localized delivery of drugs is a key advantage of 3D-printed drug delivery systems. By embedding drugs directly into the scaffold or creating drug-eluting coatings, 3D printing allows for targeted treatment of specific wound areas. This approach is particularly useful for delivering antibiotics to prevent or treat infections at the wound site, anti-inflammatory drugs to reduce inflammation and promote healing, and growth factors to stimulate cellular activities and tissue regeneration. For instance, 3D-printed wound dressings with embedded antibiotics have demonstrated significant efficacy in reducing bacterial load and enhancing wound closure [46]. Similarly, anti-inflammatory agents integrated into the scaffold matrix can help manage chronic inflammation and improve the healing process [47]. The precision and customization offered by 3D printing enable the development of highly effective localized drug delivery systems.

The integration of 3D printing with advanced drug delivery technologies offers a promising approach to improving wound care. By enabling the development of customized, drug-loaded scaffolds and leveraging supramolecular chemistry, these systems enhance drug delivery efficiency and support effective wound healing [48].

Challenges and limitations

The integration of 3D printing technology into wound care and regenerative medicine presents several challenges and limitations that must be addressed to fully realize its potential. These challenges span technical, biological, economic, regulatory, and ethical domains.

Technical and Biological Challenges

One of the primary technical challenges in 3D printing for wound care is ensuring the vascularization of the printed constructs. Effective wound healing requires adequate blood supply to support the delivery of nutrients and the removal of waste products. However, creating functional blood vessels within 3D-printed scaffolds remains a significant challenge [49]. Researchers are exploring various strategies, such as incorporating vascular channels or using bioactive factors to promote angiogenesis, but achieving fully functional vascular networks in printed tissues is still an area of ongoing research.

Immune response is another critical biological challenge. The introduction of foreign materials into the body can elicit an immune response, potentially leading to inflammation or rejection of the 3D-printed constructs [50]. The biocompatibility of printing materials is essential to minimize adverse immune reactions. Researchers are focusing on developing materials that are not only biocompatible but also capable of mimicking the natural ECM to reduce immune responses and enhance integration with host tissues.

Degradation of materials is also a concern, particularly for biodegradable scaffolds. The rate at which these materials degrade can impact their performance and the healing process. If degradation occurs too quickly, it may compromise the structural integrity of the scaffold before adequate tissue regeneration has occurred. Conversely, if degradation is too slow, it may impede natural tissue formation and prolong the presence of foreign material in the wound site [51].

Economic and Regulatory Challenges

The economic aspects of 3D printing in wound care include the high costs associated with advanced printing technologies, materials, and the development of customized products. The initial investment in 3D printing equipment and the ongoing costs of materials and maintenance can be substantial, which may limit accessibility and widespread adoption [52].

Regulatory challenges involve ensuring that 3D-printed wound care products meet safety and efficacy standards before they can be approved for clinical use. The regulatory pathway for these products can be complex, as they must comply with stringent requirements set by health authorities. The approval process often involves extensive preclinical and clinical testing to demonstrate the safety and effectiveness of the products [53]. Streamlining these regulatory processes while maintaining rigorous standards is crucial for the successful integration of 3D printing technologies into clinical practice.

Ethical Considerations and Patient Safety Concerns

Ethical considerations in the use of 3D printing for wound care include issues related to patient consent, privacy, and the potential for misuse of the technology. Patients must be fully informed about the benefits and risks associated with 3D-printed products and provide consent before treatment [54]. Additionally, there are concerns about the long-term safety and effectiveness of new technologies, and it is essential to ensure that these products do not compromise patient well-being.

Patient safety concerns also involve the potential for adverse effects resulting from the use of new materials or technologies. Continuous monitoring and post-market surveillance are necessary to identify and address any issues that may arise after the introduction of 3D-printed products into clinical settings.

Addressing these challenges requires a multidisciplinary approach involving researchers, clinicians, regulatory bodies, and policymakers. By overcoming these barriers, 3D printing technology can be more effectively integrated into wound care practices, offering innovative solutions to enhance patient outcomes.

Case studies of 3D-printed dressings and wound patches

Several case studies highlight the efficacy of 3D-printed dressings and wound patches in managing chronic wounds. For instance, researchers have developed personalized wound dressings using 3D printing to fit the unique contours and sizes of chronic wounds, such as diabetic ulcers and pressure sores. These customized dressings enhance the healing process by providing a better fit, improved protection, and more effective management of wound exudate. In one case study, a 3D-printed hydrogel-based dressing was used for a patient with a severe diabetic foot ulcer, resulting in faster wound closure and reduced infection rates. Hydrogel-based dressings have shown promising outcomes in promoting wound healing, especially for chronic wounds such as diabetic foot ulcers and burns. Studies have demonstrated that hydrogel dressings, with their high moisture content, provide a moist environment that accelerates cell migration and tissue regeneration. Another case involved the use of a 3D-printed collagen-based patch for a patient with a pressure sore, which demonstrated improved integration with the surrounding tissue and enhanced healing outcomes [55].

Future directions and innovations

As 3D printing technology continues to advance, several exciting future directions and innovations are poised to enhance wound healing and regenerative medicine. These innovations include the integration of 4D printing technologies, the incorporation of bioelectronics and integrated sensors, and the development of personalized and on-demand wound healing therapies [56].

Integration of 4D Printing Technologies in Wound Healing

The emerging field of 4D printing adds a new dimension to traditional 3D printing by incorporating the factor of time into the fabrication process. 4D printing involves the use of smart materials that can change shape or properties in response to environmental stimuli, such as temperature, pH, or moisture [57,58]. These materials, known as shape-memory polymers or self-healing hydrogels, offer the potential to create dynamic wound care products that adapt to the evolving needs of the wound environment. For instance, 4D-printed wound dressings could respond to changes in moisture levels by adjusting their permeability or releasing therapeutic agents in a controlled manner [59]. This ability to adapt in real-time could significantly improve wound management and accelerate the healing process. Ongoing research initiatives include the development of 4D-printed dressings that release anti-inflammatory drugs in response to changes in the wound’s pH levels, as well as studies focused on creating smart scaffolds for tissue regeneration that dynamically reshape in response to thermal or biological signals. Additionally, research is underway to create self-adjusting wound patches that adapt their stiffness and porosity based on the surrounding tissue conditions, promoting better integration with the wound bed [60-63].

Potential for Bioelectronics and Integrated Sensors in Wound Monitoring and Treatment

The integration of bioelectronics and sensors into 3D-printed wound care products represents a major innovation in wound monitoring and treatment. Bioelectronic devices can be embedded within 3D-printed scaffolds or dressings to monitor various wound parameters, such as temperature, pH, and glucose levels, providing valuable data for managing chronic wounds [64]. For example, smart wound dressings with embedded sensors can track the progression of wound healing and detect signs of infection or complications in real-time. This information enables more precise and timely interventions, potentially improving patient outcomes. Additionally, bioelectronic systems can facilitate the delivery of electrical stimuli or low-frequency ultrasound to promote tissue regeneration and accelerate wound healing [65].

Emerging Concepts of Personalized and On-Demand Wound Healing Therapies

The concept of personalized medicine is gaining traction in wound care, with 3D printing enabling the development of customized and on-demand wound healing therapies. Personalized approaches involve tailoring treatment strategies to the individual characteristics of each patient, such as their unique wound morphology, tissue type, and healing needs [66]. 3D printing allows for the creation of patient-specific wound care products, including custom-sized dressings and scaffolds that match the exact dimensions and requirements of the wound. Moreover, on-demand therapies, where wound care products are manufactured in real-time based on immediate patient needs, offer the potential for rapid response and adaptation [67]. For instance, a 3D printer could produce a customized wound dressing or scaffold on-site during a clinical visit, ensuring a perfect fit and optimal therapeutic effect [68].

The future of wound healing and regenerative medicine is bright with these innovative approaches. The integration of 4D printing, bioelectronics, and personalized therapies promises to revolutionize wound care by offering more effective, adaptive, and patient-centric solutions. Continued research and development in these areas will drive advancements and contribute to improved clinical outcomes in wound management [69,70].

Future Possibilities and Directions for Research in the Field

Looking ahead, several exciting possibilities and research directions are emerging within the field of 3D printing for wound care. The incorporation of 4D printing technologies promises to revolutionize wound management by enabling the creation of smart materials that respond dynamically to environmental changes. These adaptive materials could enhance the functionality of wound dressings and scaffolds, providing real-time adjustments to support the healing process. Additionally, the integration of bioelectronics and sensors into 3D-printed products offers the potential for advanced wound monitoring and intervention, allowing for more precise and personalized treatment strategies. Future research is also likely to focus on optimizing material properties, improving scalability, and exploring new applications for personalized and on-demand wound care solutions.

Despite the significant progress made in 3D printing technologies for wound healing, several gaps remain in the current research. One major limitation is the lack of standardized protocols for the design, fabrication, and clinical application of 3D-printed wound care products, which hinders their widespread adoption. Additionally, while 3D printing has demonstrated promise in creating complex structures, the long-term biocompatibility and stability of these materials in vivo require further investigation. Another gap lies in the limited understanding of the interactions between 3D-printed materials and the wound microenvironment, which could influence healing outcomes. Furthermore, while personalized wound care is a major focus, research on fully customized, patient-specific solutions that integrate real-time monitoring and adaptive functionality is still in the early stages. Future studies should address these challenges by focusing on improving material properties, enhancing scalability, and validating clinical outcomes through large-scale trials.

## Conclusions

3D printing has revolutionized wound care by enabling the creation of highly customized and complex products, such as scaffolds and dressings, tailored to individual wound characteristics. Using advanced materials like biodegradable polymers and bioactive substances, these innovations support tissue regeneration, improve biocompatibility, and allow controlled drug release. Integrating 3D printing with drug delivery systems has further enhanced treatment precision, enabling localized therapies and effective management of chronic wounds.

As research progresses, the clinical adoption of 3D-printed wound care products promises to transform personalized medicine by providing tailored solutions that improve patient outcomes and reduce healthcare costs. The customization capabilities of this technology align with the principles of personalized care, paving the way for widespread use in routine clinical practice and setting a new standard in wound management.
